# Applications of Machine Learning in Food Safety and HACCP Monitoring of Animal-Source Foods

**DOI:** 10.3390/foods14060922

**Published:** 2025-03-08

**Authors:** Panagiota-Kyriaki Revelou, Efstathia Tsakali, Anthimia Batrinou, Irini F. Strati

**Affiliations:** Department of Food Science and Technology, University of West Attica, Agiou Spyridonos, 12243 Egaleo, Greece; etsakali@uniwa.gr (E.T.); batrinou@uniwa.gr (A.B.); estrati@uniwa.gr (I.F.S.)

**Keywords:** neural networks, supervised learning, unsupervised learning, risk assessment, dairy products, meat, fish

## Abstract

Integrating advanced computing techniques into food safety management has attracted significant attention recently. Machine learning (ML) algorithms offer innovative solutions for Hazard Analysis Critical Control Point (HACCP) monitoring by providing advanced data analysis capabilities and have proven to be powerful tools for assessing the safety of Animal-Source Foods (ASFs). Studies that link ML with HACCP monitoring in ASFs are limited. The present review provides an overview of ML, feature extraction, and selection algorithms employed for food safety. Several non-destructive techniques are presented, including spectroscopic methods, smartphone-based sensors, paper chromogenic arrays, machine vision, and hyperspectral imaging combined with ML algorithms. Prospects include enhancing predictive models for food safety with the development of hybrid Artificial Intelligence (AI) models and the automation of quality control processes using AI-driven computer vision, which could revolutionize food safety inspections. However, handling conceivable inclinations in AI models is vital to guaranteeing reasonable and exact hazard assessments in an assortment of nourishment generation settings. Moreover, moving forward, the interpretability of ML models will make them more straightforward and dependable. Conclusively, applying ML algorithms allows real-time monitoring and predictive analytics and can significantly reduce the risks associated with ASF consumption.

## 1. Introduction

Foods derived from animal sources are rich in essential micronutrients that are crucial to the human diet and difficult to obtain from a plant-based diet [1]. Animal-Source Foods (ASFs) are vulnerable to biological, chemical, and physical hazards that compromise food safety and pose risks to human and animal health [2,3]. Food supply chains require strict measures to ensure food safety [4,5,6]. Framework conditions for food security deal with these concerns by systematically identifying these concerns, adhering to legal standards and implementing risk management measures [7,8]. The Hazard Analysis Critical Control Point (HACCP) management system is a fundamental component of food safety frameworks due to its systematic hazard identification and control of food safety risks [9]. The HACCP system covers stages from primary production to initial food preparation and processing, as well as subsequent handling after production [10]. Through its focus on Critical Control Points, the HACCP system enables proactive food safety management, reducing dependence on the testing of the final products [11]. The HACCP system’s effectiveness relies on manual inspections, which require extensive labor and are prone to human error [12]. The growing complexity of the global food supply chain poses issues for traditional HACCP monitoring approaches [13].

Technological developments in machine learning (ML) enable the automation of monitoring processes and enhance predictive capabilities, leading to improved food safety measures [14]. By analyzing extensive datasets, ML algorithms can detect microbial contamination, predict spoilage, and improve the traceability optimization of ASF supply chains [14,15]. ML differs from traditional methods by enabling immediate hazard detection and predictive analytics [16,17]. The enhancement of HACCP procedures through ML stems from its ability to process and analyze extensive data collected from sensor-based techniques that detect irregularities that manual inspections might miss [15,18,19]. ML enhances food safety compliance through anomaly detection (AD) and predictive modeling to maintain regulatory standards and decrease manual monitoring efforts [20,21]. The integration of ML into HACCP systems shows great potential but remains under development as multiple challenges continue to exist. ML has succeeded in food processing and distribution sectors but remains largely unimplemented at primary production stages, such as monitoring feed quality and farm animal health [22,23,24]. Current food safety frameworks reveal a critical weakness in their dependence on manual assessments during the initial stages of ASF production. To bridge this crucial gap, food safety frameworks require automated systems that leverage ML to reduce human input while maintaining high accuracy [25,26]. Automation poses substantial technological obstacles, such as real-time data integration requirements, high computational demands, and the need for systems to work across different food production stages [27,28]. The main challenge is obtaining standardized data while ensuring its availability. ML models deliver accurate results when trained on high-quality, well-labeled datasets. ML model performance suffers from inconsistent data quality, which simultaneously generates issues related to bias transparency and regulatory compliance in decision-making processes [29].

To encourage the adoption of ML within food safety frameworks and the HACCP system and to provide insight for future research, the present review explores the use of ML applications to improve food safety and HACCP monitoring in ASFs. While various studies have explored ML applications in food safety [25,28,30,31,32] and animal farming [22,33], few have explicitly linked ML to food safety in ASFs [34]. In this study, ASFs include meat derived from pigs and poultry, ruminants (including cattle and sheep), dairy products, eggs, fish, and related processed food items. The literature review utilized multiple databases, including Scopus, Web of Science, PubMed, and JSTOR. The search strategy used predetermined keywords (such as “machine learning”, “supervised learning”, “deep learning”, “neural networks”, and “unsupervised learning”) along with ASF-related terms (such as “dairy”, “milk”, “cheese”, “meat”, “fish”, and “eggs”). Additionally, the search strings included terms like “food safety” and “HACCP”.

## 2. Feature Selection and Feature Extraction

In ML and data science, high-dimensional data processing presents many challenges for researchers. Using high-dimensional data to train ML algorithms may lead to an overfitted model that identifies noise and erratic variations as learned concepts, making it function inadequately on unseen data [35]. Dimensionality reduction is essential to avoid overfitting because it minimizes computational costs, improves model interpretations, and reduces redundancy by simplifying models [36]. Feature selection and feature extraction have one primary distinction: feature selection retains a subset of the original features, while feature extraction creates a completely new subset [37].

### 2.1. Feature Selection

Feature variable or attribute selection involves selecting a subset of distinct features for developing ML and data science application models. Removing unnecessary or insignificant features simplifies a model and speeds up the training of ML algorithms. Identifying the relevant and optimal subset of chosen features can reduce the risk of overfitting when building ML models. Feature selection is a fundamental concept in ML and significantly influences the effectiveness of the target model [38].

Feature selection algorithms encompass various techniques, including Analysis of Variance (ANOVA), Recursive Feature Elimination (RFE), Genetic Algorithms (GA), stepwise regression, Least Absolute Shrinkage and Selection Operator (LASSO), and the Boruta algorithm. ANOVA has been utilized to extract features from the Raman spectra for the Convolutional Neural Networks (CNN) [39]. The RFE algorithm with cross-validation has been employed to select the optimal input feature sets from datasets associated with milk quality for predicting subclinical mastitis [40,41]. Alshejari et al. [42] used seven feature selection algorithms to predict total viable counts through multispectral imaging. These included RFE, GA, LASSO, relative importance from linear regression, Partial Least Squares (PLS), and the Boruta algorithm. The Boruta algorithm has also been used to select features in near-infrared spectra [43].

### 2.2. Feature Extraction

In ML-based models, feature extraction enhances understanding of the data, boosts prediction accuracy, and reduces computational cost and training time. In the feature extraction process, the original set of features is reduced, creating a new set of features. Data obtained from spectroscopic techniques are high-dimensional with multicollinearity issues [44]; therefore, feature extraction is frequently applied. Principal Component Analysis (PCA) is commonly used as a dimensionality-reduction technique to extract a lower-dimensional space and create new components, known as principal components [45,46]. PCA has been applied by Lu et al. [19] for the feature extraction of the Raman spectra for monitoring the antibiotic ofloxacin in meat. Another feature extraction algorithm is Competitive Adaptive Reweighted Sampling (CARS), based on Monte Carlo sampling and PLS regression. The CARS algorithm has been applied by Feng et al. [47] in Raman spectra to identify dairy fraud. Two pre-trained CNNs, SqueezeNet and InceptionV3, have been used by Yasin et al. [48] for feature extraction on an image dataset.

## 3. Machine Learning Algorithms

ML involves exploring and developing mathematical models and algorithms that enable computers to learn from the provided input data. In this context, learning is defined as the process (based on a learning algorithm) of translating the input of experience (such as historical data) into the output of expertise (e.g., classification and prediction) [49,50]. Supervised ML algorithms are primarily utilized for applications relating to food safety [25,30,32]. Supervised ML algorithms analyze labeled datasets and training examples to derive a function. Supervised learning is typically employed when specific goals are defined based on a set of inputs, taking a task-driven approach. The prevalent supervised tasks are classification (which separates the data) and regression (which fits the data). Classification concerns educating the computer program on a training dataset, which enables it to categorize the data according to the class labels [37,51]. There are several established supervised ML algorithms, including Support Vector Machine (SVM), Naive Bayes (NB), Decision Trees (DT), K-Nearest Neighbors (KNN), Logistic Regression (LR), Random Forest (RF), Discriminant Analysis (DA), and Gradient Boosting (GB). The food safety monitoring process frequently employs unsupervised learning, which can analyze unlabeled datasets that are exclusive to human assistance. Additionally, unsupervised AD can identify atypical data points (that are notably different from the majority of data in a dataset) by using historical data to determine which readings fall within the acceptable range [52]. This approach is critical in maintaining the quality and security of data across numerous domains (including food safety).

### 3.1. Naive Bayes

The NB algorithm applies Bayes’ theorem while assuming that features are conditionally independent once the class label is known. The robust assumption enables NB to function with little training data when estimating parameters, which results in computational efficiency [53]. The independence assumption of the NB algorithm can hinder its performance especially when features exhibit high correlation. NB classifiers come in multiple variations such as Gaussian, Multinomial, Complement, Bernoulli, and Categorical [54].

**Figure 1:**
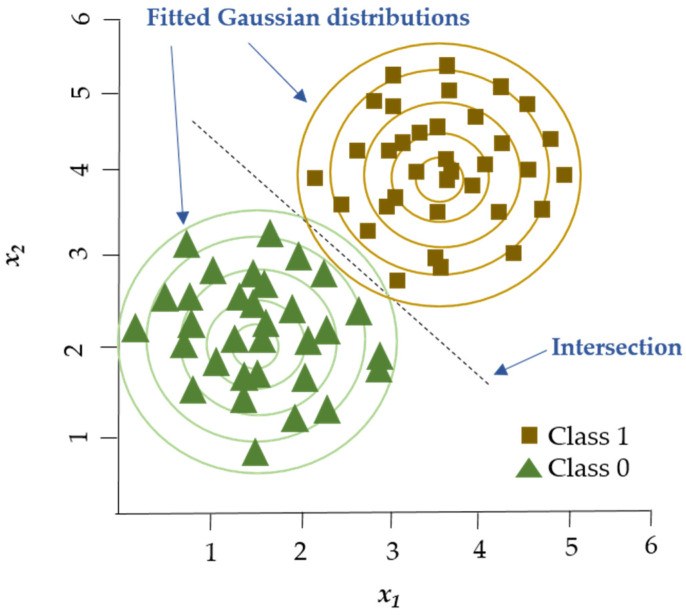
GNB algorithm. GNB classifier models two Gaussian distributions corresponding to the labeled groups in the dataset. The decision boundary is established at the location where the probability densities of the two groups are equal. Adapted from Shyrokykh et al. [55].

The Gaussian Naïve Bayes (GNB) algorithm (Figure 1) has been utilized for multiple classification tasks including animal disease prediction. Satoła and Satoła [40] applied the GNB algorithm for the detection of subclinical mastitis in dairy cows. The research utilized milk sample data from dairy farms which included somatic cell count (SCC), milk composition parameters such as fat and protein content along with environmental factors. The dataset served as the benchmark for evaluating GNB’s performance in comparison to ensemble methods including bagging, boosting, stacking, and super-learners. GNB provided an easy-to-understand method, but its effectiveness depended on the distribution of features in the data and the normality assumption for continuous variables.

### 3.2. K-Nearest Neighbors

**Figure 2:**
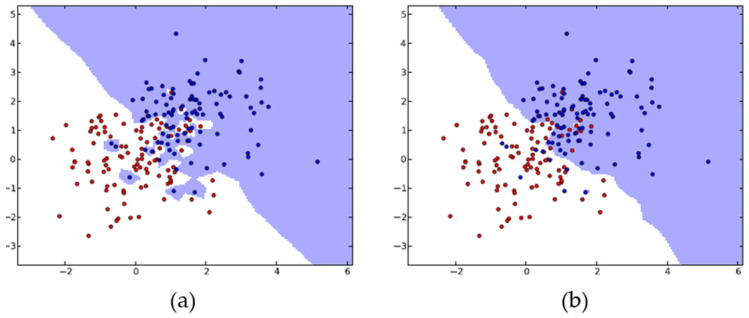
KNN algorithm with *K* = 1 (**a**) and *K* = 20 (**b**). The KNN generalizes for larger *K* values, while it tends to overfit for small numbers of neighbors. Adapted from Kramer [56].

The KNN algorithm employs proximity to classify new data points (e.g., Euclidean distance function) and is applied for both regression and classification. Classification is calculated from a simple majority vote of the KNN of each separate point. The KNN algorithm’s accuracy relies on the data’s quality and is relatively resistant to noisy training data. An important issue that must be considered in this algorithm is the selection of the optimal number of neighbors *K* (Figure 2) [56].

The KNN algorithm has been used in spectroscopic data to predict fish quality and safety [57,58,59], in images datasets to evaluate fish freshness [48], in identifying foodborne pathogens and spoilage bacteria in milk [60], and to predict subclinical mastitis in dairy cows [40]. In these studies researchers adapted the KNN algorithm by implementing customized preprocessing and optimization procedures to improve classification accuracy. The performance of the dataset improved through feature engineering methods like PCA, normalization, and feature selection which reduced dimensionality and enhanced data representation. The common approach to measuring the similarity between data points used Euclidean distance as the primary metric, although some studies tested Manhattan and cosine similarity depending on the dataset characteristics. Through cross-validation, researchers determined the best number of neighbors *K*, which maintained a balance between underfitting and overfitting.

### 3.3. Discriminant Analysis

**Figure 3:**
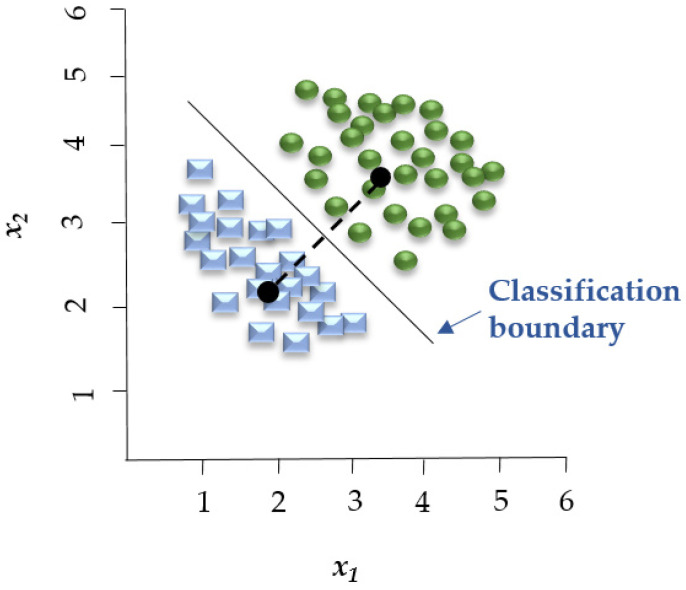
LDA establishes a linear boundary that separates the groups, effectively dividing the space between the centroids of these groups. Adapted from Adams [66].

Linear Discriminant Analysis (LDA) is a linear decision boundary classifier generated by fitting class conditional densities to the data and subsequently applying Bayes’ rule (Figure 3). As an extension of Fisher’s Discriminant Analysis, LDA reduces the dimensionality of a given dataset to lower the model’s complexity and associated computational costs [61]. Based on the assumption that all categories share the same covariance matrix, the LDA model associates each category with a Gaussian density. LDA has similar characteristics to regression analysis and ANOVA, which represent each dependent variable as a linear combination of features [37,61]. LDA has been employed in spectroscopic data to assess the quality of fish [59] and detect adulteration in beef [62].

Another Discriminant Analysis algorithm is Partial Least Squares Discriminant Analysis (PLS-DA), which combines Partial Least Squares Regression (PLSR) with Discriminant Analysis. PLSR is utilized for both prediction and dimension reduction. It facilitates the analysis of overlapping absorption peaks derived from samples containing multiple components [63]. PLS-DA combines dimensionality reduction and Discriminant Analysis into a single algorithm for modeling high-dimensional data. Furthermore, PLS-DA is more flexible than LDA as it does not require the data to follow a specific distribution [64].

PLS-DA has been used on spectroscopic data to classify milk from cows with subclinical mastitis [65] and identify the freshness grades of sea bass [39]. The two studies used preprocessing methods, including normalization and baseline correction, alongside feature extraction, to remove noise from the spectra dataset. Through PLS-DA dimensionality reduction, data simplification was achieved by selecting the essential features from complex high-dimensional spectral data. The dimensionality reduction process enabled the PLS-DA algorithm to concentrate on essential patterns that were relevant to the classification task. PLS-DA is especially useful in analyzing complicated spectral data since it works without requiring data distribution assumptions.

### 3.4. Support Vector Machine

**Figure 4:**
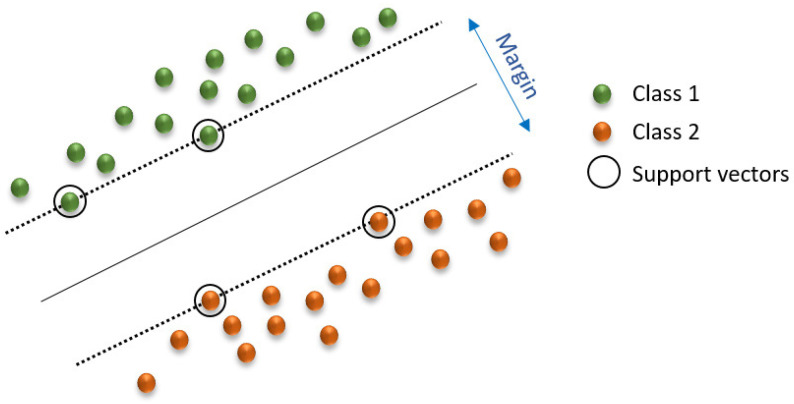
Linear SVM model. Adapted from Ahmetoglou and Das [67].

SVM can be used for classification and regression. In high- or infinite-dimensional space, an SVM constructs a hyperplane (or a set of hyperplanes) to achieve a strong separation of groups (Figure 4) [67]. Typically, this separation occurs because the greater the margin, the smaller the generalization error of the classifier. Therefore, it is effective in high-dimensional spaces and behaves differently depending on various functions (radial basis function, polynomial, etc.) [68].

The SVM algorithm is used in various applications to assess food safety and quality due to its effectiveness in managing high-dimensional data classification tasks. Wang et al. [60] utilized the SVM algorithm in a single-stranded DNA sensor array to detect multiple foodborne pathogens and spoilage bacteria found in milk. The algorithm processed sensor-generated information, which classified bacterial species according to their unique hybridization patterns. Feng et al. [47] utilized SVM with Raman spectroscopy for dairy fraud detection by training models to distinguish between authentic and contaminated dairy products based on spectral changes. Talari et al. [69] applied SVM within a data-driven system designed for hazard prioritization to classify dairy product risks from microbial and chemical sources using open-source food safety databases. In spectroscopic applications, Currò et al. [43] utilized SVM to classify histamine concentrations in tuna through near-infrared (NIR) spectral data analysis. Similarly, Kashani Zadeh et al. [59] used multi-mode spectroscopy combined with fusion-based AI systems for the assessment of fish freshness across various supply chain nodes, with SVM playing an essential role in freshness detection. Ninh et al. [57,58] employed SVM and NIR spectroscopy to determine the levels of histamine and urea in fish samples. Their research demonstrated the algorithm’s effectiveness in processing complex spectral data for assessing food quality. The researchers optimized SVM through feature selection and kernel tuning methods, such as radial basis function and polynomial kernels, combined with cross-validation. This approach led to precise classification results in sensor-based and spectral food safety applications and database-driven solutions.

### 3.5. Random Forest

**Figure 5:**
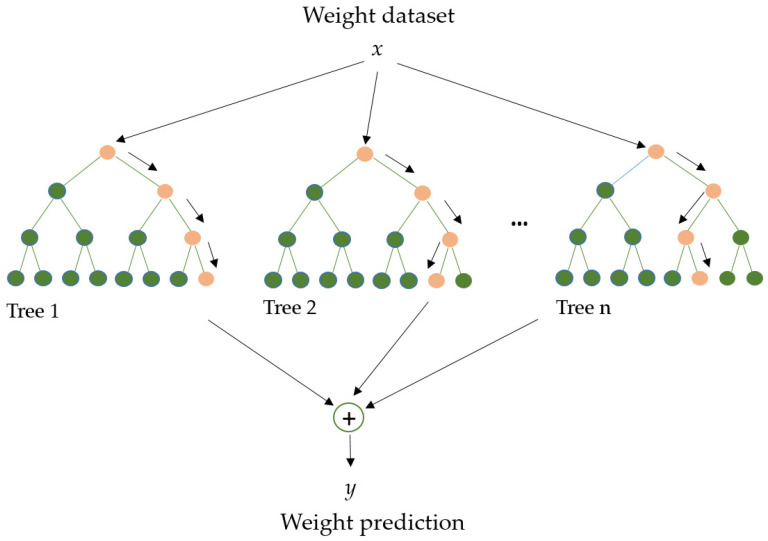
Random Forest model. Adapted from Yang et al. [72].

RF (Figure 5) is an algorithm employed in various ML and data science areas. It employs a parallel ensemble technique that fits numerous Decision Tree classifiers in parallel (across diverse dataset sub-samples) and uses majority voting (or averages) to arrive at an outcome. This approach minimizes overfitting while increasing prediction accuracy. As a result, the multiple Decision Tree RF learning model is acknowledged as more accurate than a model based on a single Decision Tree. Bootstrap aggregation (bagging) and random feature selection are combined in this method to produce a set of Decision Trees with controlled variation, making it appropriate for both classification and regression [70,71].

Researchers applied the RF algorithm to sensor-collected data for spoilage detection, as shown by Surjith et al. [73] and Wang et al. [60]. The study by Surjith et al. [73] employed an ensemble model combining RF-CNN-GRU techniques for beef quality evaluation. In ensemble modeling, RF ranks feature importance and manage high-dimensional data while acting as a base learner. RF processed biosensor signal data in milk bacterial identification through a DNA sensor array [60] using bootstrapped Decision Trees for classifying multiple foodborne pathogens and spoilage bacteria.

Cao et al. [74] employed RF to colorimetric sensor data to evaluate fish freshness. The sensor response data revealed important colorimetric features that helped identify patterns linked to fish freshness. RF constructed multiple Decision Trees to analyze how sensor color changes relate to total volatile basic nitrogen levels, which indicate spoilage. Through bootstrapping and feature randomness, the algorithm reduced overfitting while improving generalization to achieve a reliable classification of freshness states.

Currò et al. [43] applied RF to improve feature selection for histamine level classification in frozen-thawed tuna fish. The Boruta algorithm enabled RF to select important wavelengths from NIR spectral data while removing irrelevant and noisy features. The classification accuracy improved significantly because this step concentrated on spectral bands which showed the strongest correlation with histamine concentration. The analysis showed that selected wavelengths existed mainly in the visible and near-infrared regions which demonstrated interactions between histamine and the molecular structure of tuna. RF optimized the histamine detection process by decreasing data dimensionality and boosting predictive performance.

Several studies used the RF algorithm for food safety and microbiological risk prediction. Al et al. [75] used RF to predict *E. coli* growth rates in raw ground beef. Date were preprocessed using standard normal variate transformation followed by training of the RF model with temperature and time inputs to predict bacterial populations.

The RF algorithm was utilized to develop an early warning system for predicting *Salmonella* outbreaks in northwestern Italy [76]. The dataset contained information about human infection cases together with food safety audit results and spatial-temporal prediction factors. The RF algorithm demonstrated its ability to handle high-dimensional data while utilizing bootstrapping techniques to achieve strong generalization performance and, by ranking feature importance, it proved to be an effective tool for food safety monitoring and microbiological risk assessment across these studies.

The classification of milk into distinct quality grades (low, medium, high) relied on physicochemical parameters using RF, as analyzed by Bhavsar et al. [77]. The preprocessing stage required label encoding for categorical features and Min–Max normalization of numerical features while also confirming the absence of missing data points. Through the application of bagging techniques and feature importance evaluation, the model determined pH and fat content as the most critical indicators of milk quality.

### 3.6. Decision Trees

**Figure 6:**
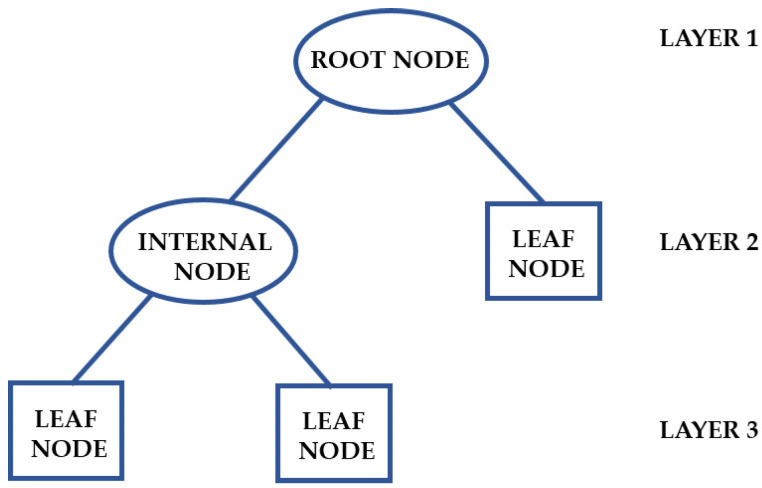
Structure of a DT. Adapted from Chiu et al. [79].

Problems involving classification and regression can be resolved by applying a DT algorithm, which observes data about an instance and generates an accurate report of its target value. Creating a tree that represents the complete dataset to minimize the error rate for each leaf or producing a result for each one are the fundamental ideas behind a DT. A decision rule (represented by a branch) and an internal node determine the structure of a DT. A distinct leaf node represents each result (Figure 6). A DT has a topmost node (root node), which partitions the tree via the feature value and employs recursive partitioning to create a clear diagram with a logical structure [78].

Talari et al. [69] used the DT model to categorize food safety alerts regarding chemical contaminants in dairy products, implementing recursive splits based on crucial predictors. The model utilized classification and regression trees to determine decision points, including reference dose, substance amount, notification type, product category, and contaminant type, to differentiate between “serious” and “non-serious” alerts. The tree was pruned to six levels to minimize overfitting and enhance generalization. Satoła and Satoła [40] utilized DT as a classification model to predict subclinical mastitis in dairy cows by analyzing milk performance data. The model segmented the dataset into smaller subsets through a recursive process of selecting the most relevant features at each step. To avoid overfitting, the tree was pruned by limiting its depth, and the training set was split 80:20 to prevent data leakage. Ninh et al. [57,58] utilized the DT model in NIR spectroscopy data. The model classified fish samples into “Safe” and “Unsafe” categories based on urea and histamine content in fish samples by organizing the information into a hierarchical tree structure where nodes acted as decision rules based on specific wavelengths’ spectral absorbance values. The algorithm identified spectral features by recursively splitting the data according to key wavelengths and absorbance values using specific contamination thresholds such as Safe (<100 ppm) and Unsafe (>100 ppm) for histamine classification. Pruning methods were used to reduce overfitting, while the synthetic minority over-sampling technique generated additional synthetic instances of minority class samples to tackle class imbalance. Internal tail NIR spectra provided the DT model with a moderate classification accuracy of 87.2% for histamine detection and yielded comparable results for urea detection.

### 3.7. Gradient Boosting

**Figure 7:**
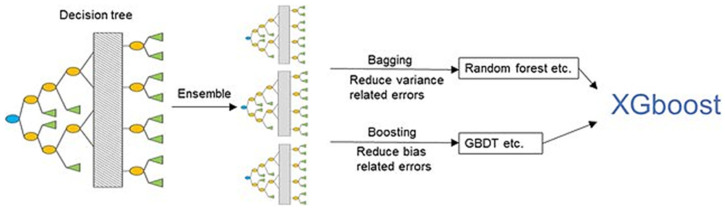
XGBoost model. From Jiang et al. [83].

GB is an example of an ensemble learning algorithm that creates a definitive model by implementing a series of individual models (such as DT). In this approach, the gradient is employed to minimize the loss function (similar to how neural networks utilize gradient descent to optimize weights) [80,81]. Extreme Gradient Boosting (XGBoost) (Figure 7) is a variant of GB that considers detailed approximations when establishing the most effective model. By calculating the second-order gradients of the loss function to reduce loss and employing advanced regularization (L1 and L2), XGBoost can minimize overfitting, improve model generalization, and enhance performance. Additionally, this approach can be interpreted rapidly and efficiently to manage large datasets [80,82].

Another form of GB is the Light Gradient Boosting Machine (LightGBM), a tree-based ensemble method designed to overcome the efficiency and scalability challenges faced when using XGBoost with high-dimensional input features and large datasets. LightGBM prioritizes computational efficiency while maintaining acceptable levels of accuracy. In this context, the term “light” indicates that this algorithm delivers faster performance compared to other Gradient Boosting methods (such as XGBoost, which requires extensive training when handling large datasets) [84]. Category boosting (CatBoost) is specifically designed to handle categorical variables in contrast to other ML algorithms that necessitate converting categorical variables into a numerical format using techniques like one-hot encoding. CatBoost can process these variables directly, streamlining the data preparation process and enhancing performance.

Feng et al. [47] used XGBoost and LightGBM algorithms to identify dairy brands and measure their fat content. XGBoost and LightGBM analyzed Raman spectral data through high-dimensional feature pattern recognition while concentrating on Raman shifts that identify dairy products. Gradient descent-based optimization guided the training of models to progressively diminish misclassification errors and preserve interpretability. XGBoost demonstrated marginally better performance than LightGBM in discerning complex spectral variable interactions, while both models maintained a classification accuracy above 90%. Yan et al. [85] utilized XGBoost Regression for analyzing *Escherichia coli* O157:H7. The model refined weak learners using gradient descent optimization in iterative steps to reduce residual errors. The signal intensity at 1335 cm^−1^ in the Raman spectral data was the input feature that established a correlation with bacterial concentration. XGBoost Regression provided better predictive results than linear regression. Son et al. [86] utilized XGBoost, LightGBM, and CATboost to evaluate hyperspectral imaging data for predicting nitrate levels in pork sausages. Hyperspectral images spanning the 1000–2500 nm range served as input for the models. The 1080 and 1280 nm wavelengths were selected as markers for nitrite effects on protein matrix interactions and water-binding capacity. The sequential boosting approach applied in this study reduced residual errors and enhanced predictive accuracy.

### 3.8. Deep Learning

Deep learning, also known as a deep neural network, is a category of representational learning that enables the extraction of features from raw data for detection, classification, or regression, while refining multi-level representations through Artificial Neural Networks (ANNs). Unlike traditional ML, which necessitates significant human collaboration to yield results, deep learning can learn from its errors [87]. ANNs consist of a series of computational algorithms, including activations and weights that transform data from input to output, designed to uncover underlying relationships in a dataset. The self-learning capability of ANNs depends on loss, Back Propagation, and gradient descent algorithms. Training a neural network may involve manual supervision, unsupervised learning, or a blend of both methods, and can be tailored based on the availability and characteristics of the training data [88].

There are two principal classes of ANNs: Feedforward Neural Networks and Recurrent/Feedback Networks. Feedforward Neural Networks are founded on biological neural networks and consist of basic neuron-like computational units organized in layers. In these systems, the input data are circulated throughout the network until received at the outputs. When this operation occurs as expected, it serves as a classifier and no feedback is presented in these layers [89]. The primary difference between Feedforward and Recurrent/Feedback neural networks is that Feedforward networks convey the data forward (from input to output), whereas Recurrent networks possess a feedback loop that enables the data to be transferred back to the input and fed forward again for additional processing before arriving at a final output. Types of Feedforward Neural Networks include Single-Layer Perceptron (SLP), Multilayer Perceptron (MLP), Radial Basis Neural Networks (RBNN), and Extreme Learning Machines (ELM) [90].

Research by Jia et al. [91,92] revealed that feed-forward neural networks can process sensor data, enabling the concurrent tracking of multiple pathogens. Utilizing a standard rectified linear unit activation function accelerated learning and mitigated vanishing gradients in the hidden layers. In the output, the SoftMax activation function produced bacterial classification probability distributions. The use of the Adam optimizer, combined with the cross-entropy loss function, improved both convergence speed and classification accuracy. A five-fold cross-validation strategy was employed to assess model robustness.

The MLP neural networks have been used in sensor-obtained data for quality monitoring and traceability in the dairy chain [93]. The model processed 11 milk parameters to identify deviations from standard milk quality levels. The MLP architecture consisted of an input layer with 9 features, two hidden layers with 20 neurons each, and an output layer with 4 nodes, corresponding to the identified anomaly classes. Each node in the hidden layers utilized the sigmoid activation function, whereas the output layer employed SoftMax activation to classify milk samples into their corresponding anomaly groups. The model underwent training through a Back Propagation algorithm with gradient descent, implementing an early stopping technique to avert overfitting. Daily standardization of data countered sensor drift, and a grid search method fine-tuned the number of neurons in each layer by minimizing the mean-square error. Cui et al. [94] used RBNN and ELM to determine seafood freshness. The RBNN demonstrated high performance by mapping complex nonlinear relationships in data with strong global approximation power while avoiding local minima traps. The activation mechanism of the network based on a Gaussian function enabled efficient pattern recognition, while the careful choice of 54 neurons maintained both accuracy and computational efficiency. The ELM model achieved fast learning capabilities through random weight initialization and output weight determination via least squares computation. ELM outperforms traditional feedforward networks by optimizing the output weights through a single training step which speeds up learning and achieves high prediction accuracy.

Recurrent/Feedback Networks can process data comparable to the human brain. When in operation, recurrent networks are small in size (compared to Feed Forward Networks), can be employed in a variety of contexts, and exhibit robust computational power. Currently, there are several varieties of Recurrent/Feedback Networks including the Kohonen Self-organizing Map Networks (SOM), Hopfield Networks, Adaptive Resonance Theory (ART) Models, Long Short-Term Memory (LSTM) Networks, and CNN [89,90].

The application of LSTM and CNN architectures in computer vision has been utilized to evaluate beef quality ([95]). Image feature extraction for classification tasks was conducted by CNNs. The models employed pre-trained CNN architectures, including VGG16, ResNet50, and AlexNet, to process RGB images and spectral data, enabling them to capture hierarchical visual patterns and spectral variations. The study achieved improved classification accuracy by combining handcrafted color features from multiple color spaces with CNN-extracted deep features and further enhanced results with a Bi-LSTM-augmented fusion model, reaching an accuracy of 98.9%. CNNs have been applied to detect beef adulteration by identifying the presence of colorants and curing agents [96]. The model extracted significant spectral features from 344 to 1040 nm using AlexNet. Additionally, gradient-weighted class activation mapping indicated that the 580–600 nm region was essential for the classification of samples.

An important application of deep learning is AD, which concerns identifying samples significantly distinct from the bulk of the data and representing irregular, atypical, or unreliable observations [97]. AD plays a crucial role in instances where not all existing classes can be defined during training and is particularly compatible with food distribution and safety inspection data because most high-risk samples are atypical. Therefore, AD algorithms can facilitate the practical assessment of food safety hazards [98]. Auto-encoder (AE) is a neural network that is commonly employed for AD. This type of neural network has a symmetric structure consisting of an encoder and a decoder designed to find a compressed representation of the provided input data. This process involves finding a representation (or code) to conduct beneficial transformations on the input data, and, typically, AEs are recommended for dimensionality reduction or feature learning [99,100].

**Figure 8:**
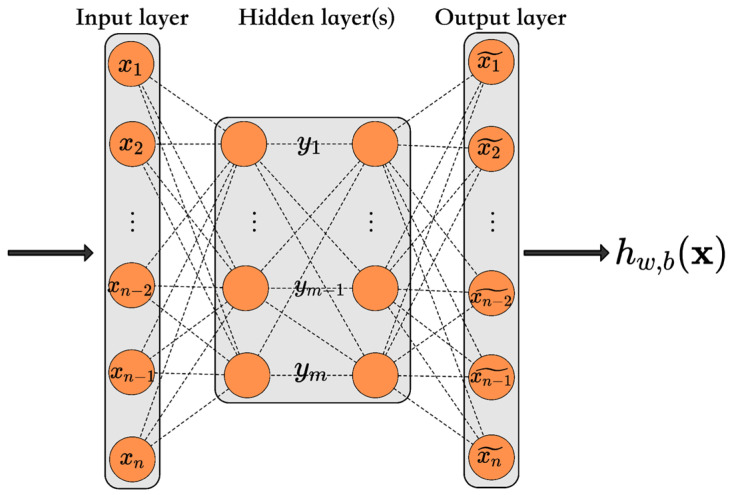
Auto-encoder. It has a symmetric structure with an encoding and a decoding phase. In the encoding phase, there is a compressed representation of the data, and in the decoding phase, the original input is reconstructed. From Zuo et al. [18].

Anomaly score-based risk early warning systems using AE neural networks (Figure 8) have been developed for food safety monitoring of HACCP procedures to detect potentially unqualified products [18]. During the reconstruction of input data, the system detected anomalies through reconstruction error analysis. The AE model analyzed 2.158 milk samples with variables including lactose levels, acidity, non-fat milk solids, fat, protein percentages, and aflatoxin concentrations. The model accurately reconstructed normal samples due to the consistent distribution of “qualified” food samples. It demonstrated significantly higher reconstruction errors for unqualified samples because these samples deviated from the normal pattern it had learned. The error measurement provided the foundation for calculating the anomaly score, which determined the sample’s risk level. The de-noising Auto-encoder variant enhanced robustness by effectively handling noisy data and incomplete datasets through the introduction of Gaussian white noise to the inputs.

## 4. Applications of Machine Learning Algorithms in Food Safety and HACCP

Table 1, Table 2 and Table 3 summarize the main applications of ML algorithms in assessing the food safety of ASFs, including risk assessment and HACCP monitoring, identification and prevention of chemical and microbiological hazards, fraud/adulteration detection, and quality assessment. Databases, various spectroscopic techniques, imaging (RGB, multispectral, hyperspectral), and sensors have been utilized as data sources for the application of ML. The literature shows that the most widely utilized algorithms are various types of neural networks (MLP, LSTM, CNN) followed by SVM and RF. Neural networks have the advantage of processing large datasets, and, therefore, they are frequently applied in imaging, sensor, and spectroscopic data. However, neural networks are difficult to interpret and slow to train. SVM and RF are simpler and more straightforward to interpret, providing the advantage of avoiding overfitting compared to other ML algorithms [49,50].

### 4.1. Food Safety Risk and HACCP Monitoring

Food safety early warning systems function in three ways: to detect any potential issues via risk analysis, to control risk in the food decision-making process, and to provide information to support improvements in food standards regulation and decision-making; therefore, a robust risk analysis model is crucial for effective warning systems [101].

To facilitate the effectiveness of an early warning system for food safety risk assessment, Zuo et al. [18] presented two unsupervised AE neural networks. This study amalgamated neural network modeling with food supply chains (in parallel with the HACCP protocols) to identify the crucial elements of risk warning and regulate risk via an in-depth hazard analysis of each testing index. The raw data used in this study concerned fresh milk, and the indicators used for the AE algorithm were acidity, nonfat milk solids, fat, lactose, protein, and aflatoxin M1 (AM1). The subsequent model attained elevated levels of accuracy (0.9954), which indicates its effectiveness in analyzing data regarding dairy products.

Utilizing large datasets from databases has become critical for assessing and addressing food safety hazards. The World Health Organization’s Global Environmental Monitoring System (GEMS) is an important database. The GEMS is a comprehensive food database that encapsulates data about chemicals (e.g., heavy metals, pesticides, persistent organic pollutants, veterinary drug residues) and biological contaminants, such as mycotoxins, biotoxins, and radionuclides [102]. The Rapid Alert System for Food and Feed (RASFF) is another important database operated by the European Union, which provides data on allergens, foodborne pathogens, heavy metals, pesticides, mycotoxins, and biotoxins [103]. Talari et al. [69] employed ML models (such as DT, RF, KNN, LDA, GNB, and SVM) to classify food safety alerts concerning the chemical and microbial contamination of dairy products using data from RASFF and GEMS databases. The results indicated that the most accurate ML models were DT and SVM (0.98) followed by RF (0.94), GNB (0.91), and KNN (0.85). The results of their exploratory data analysis ascertained the highest priority microbial hazards in dairy products: *Listeria monocytogenes*, *Escherichia coli*, *Salmonella*, *Pseudomonas* spp., *Staphylococcus* spp., *Bacillus cereus*, *Clostridium* spp., and *Cronobacter sakazakii*, and *Bacillus cereus*. Additionally, the study identified the primary chemical hazards (based on their potential negative impact on public health) as follows: nitrate, nitrite, ergocornine, 3-MCPD ester, ergosine, lead, arsenic, ochratoxin A, cadmium, mercury, followed by aflatoxins (G1, B1, G2, B2, G5, and M1).

Unsupervised AD (based on a Bayesian network algorithm) has been employed [104] to determine if the environmental, social, economic, and technological variables related to the milk supply chain can provide early warning for potential food safety hazards. The Bayesian network model was trained on the records contained in the Quality Program for Agricultural Products (KAP) database, which contains data regarding chemical hazards (e.g., dioxins, mycotoxins, pesticides, heavy metals, antibiotics) in food and animal feed from the Netherlands [105]. The total accuracy and specificity of the training and validation sets can be evaluated as good (>85%). The results indicated that the occurrence of an anomaly in certain areas (such as milk cost, feed cost, and average monthly precipitation) statistically correlates with specific food safety hazards (reported by monitoring programs) several months later.

An alternative, the HACCP-like approach, can be used to ensure safety by focusing on Points of Particular Attention (POPAs). POPAs are conditions that pose a threat to animal or human health or the management of the farm. Monitoring of POPAs may allow for better understanding and management [106]. To evaluate quality control and traceability in dairy production, Dragone et al. [93] employed a patented HACCP-analogous remote diagnostic (sensor-driven) system called BEST for the monitoring of variations in markers at CCP and POPAs (e.g., temperature, O_2_, CO_2_, redox potential, pH, conductivity, Ca^2+^, NH_4_^+^, NO_3_^−^, Cl^−^, and milk yield). The BEST system underwent daily examinations on a dairy farm regarding its ability to reliably distinguish additional indicators of safety and quality anomalies in milk production. The results were confirmed using supervised classification based on the MLP neural network and unsupervised classification (clustering) based on the SOM neural network, which spotted cows with specific characteristics.

**Table 1:** Applications of machine learning algorithms in dairy products.

Product	Purpose of Study	Data	Machine Learning Algorithm	Output	Year	Reference
Food safety risk and HACCP monitoring						
Milk	Risk control by conducting a comprehensive hazard analysis of each parameter	Protein, fat, NMS ^1^, lactose, AM1 ^2^, acidity	Unsupervised anomaly detection AE ^3^, K-Means, Isolation Forest, KNN ^4^, LOF ^5^, COF ^6^, SO-GAAL ^7^	AE achieved 0.9954 prediction accuracy	2022	Zuo et al. [18]
Milk	Hazards identification associated with an anomaly, prediction of food safety hazards	Raw milk price, number of patents related to the dairy sector, feed price, income of dairy farms, usage of antibiotics, usage of antibiotics, average temperature, average precipitation, total population, average age of dairy farmers, urban population, investment in R&D related to dairy sector, level of adoption of technology	Unsupervised anomaly detection BN ^8^	>85% total accuracy	2022	Liu et al. [104]
Milk	Early Hazard Analysis and Critical Control Points and traceability in the dairy supply chain	Temperature, O_2_, CO_2_, redox potential, pH, conductivity, Ca^2+^, NH_4_^+^, NO_3_^−^, Cl^−^, milk yield	MLP ^9^	Cows with specific characteristics were spotted	2024	Dragone et al. [93]
Dairy products	Classifying food safety alerts related to chemical and microbial contaminants	Data obtained from RASFF ^10^ WHO ^11^ GEMS ^12^ databases	MLP, DT ^13^, SVM ^14^, GNB ^15^, CNN ^16^	The highest accuracy was achieved by DT and SVM (98%)	2024	Talari et al. [69]
Microbiological hazards						
Milk and beef	Detection of *E. coli* O157:H7	Surface-enhanced Raman scattering–based lateral flow assay	XGBoost ^17^	Successfully forecast *E. coli* in samples spiked with 10 CFU/mL	2020	Yan et al. [85]
Milk	Identification of practices affecting PPC ^18^	Bacterial spoilage indicators dataset (*Pseudomonas* spp.)	RF ^19^, MMI ^20^	Factors for reducing PPC were identified (quality control, sanitation, staff training)	2021	Murphy et al. [107]
Meat, eggs, shellfish, dairy products, infant formula, etc.	Identification of parameters associated with the occurrence of *Salmonella* spp.	*Salmonella* spp. occurrence based on: product, region, and stage data	RF	Accuracy achieved 77.2%	2023	Rodríguez et al. [108]
Meat, eggs, dairy products, processed food salad, fish, etc.	Identification of potential sources of *Campylobacter* spp.	*Campylobacter* spp. occurrence based on: product, region, and stage data	RF	Accuracy achieved 83.1%	2024	Sacristán et al. [109]
Cheddar cheese	Pathogen identification	*Salmonella enteritidis*, *E. coli* O157:H7 identification by paper chromogenic array sensor dataset	DFFNN ^21^	Accuracies ranging between 72 ± 11% and 92 ± 3%	2024	Jia et al. [91]
Eggs, milk, meats, bakery products, seafood, etc.	Prevention of foodborne *Salmonella* outbreaks	Food surveillance data (month, longitude, latitude, area, food prevalence, food categories)	Tree regression, RF, GB ^22^	RF and GB (R^2^ = 0.55) outperformed the tree regression algorithm (R^2^ = 0.42)	2024	Garcia-Vozmediano et al. [76]
Milk	Identification of foodborne pathogenic and spoilage bacteria	*Escherichia coli*, *Listeria innocua*, *Salmonella enterica*, *Staphylococcus aureus*, *Shigella sonnei*, *Bacillus cereus*, *Lactococcus lactis*, *Pseudomonas fluorescens* identification by single-stranded DNA sensor array dataset	PLS-DA ^23^, KNN, RF, SVM, MLP, KAN ^24^	MLP neural networks achieved the highest accuracy at 98.4%	2025	Wang et al. [60]
Milk	Detection of *Staphylococcus aureus*	Nanogap-assisted surface-enhanced Raman scattering biosensor dataset	VCPA-PLS ^25^, RF-PLS ^26^, BOSS-PLS ^27^	BOSS-PLS achieved the best results (Rp = 0.967)	2025	Xu et al. [110]
Chemical hazards						
Milk, wheat, rice, coffee, maize	Detection of mycotoxins	Cystamine-derived carbon dot array concentration of mycotoxins	XGBoost	A 100% accuracy and mycotoxin detection at 10 pmol		Aggarwal et al. [111]
Milk	Detection of antibiotics	Optical immunosensor data concentration of antibiotics	PLSR ^28^	Detection from pg/mL to ng/mL with an accuracy of >99%	2024	Zhou et al. [112]
Milk	Detection of the antibiotic levofloxacin	Quasi-ratiometric fluorescent probe provided fluorescence images	Hierarchical clustering	Low detection limit (4.53 nM) and excellent recovery rates (101.7–103.4%) were obtained	2025	Liu et al. [113]
Milk	Detection of the antibiotics norfloxacin and ciprofloxacin	Surface-enhanced Raman scattering data	SVR ^29^, RF, XGBoost	The coefficient of determination (R^2^) was 0.996, with a detection limit of 10 ppb	2025	Liu et al. [114]
Fraud/adulteration detection						
Milk	Adulteration detection	Fat, protein, non-fat solid, total solid, lactose, relative density, freezing point depression, acidity, infrared spectra	Ensemble model of ExtraTrees and XGBoost	A 0.9924 accuracy achieved	2022	Chung et al. [115]
Milk	Fraud detection	Raman spectra	LightGBM ^30^, SVM, RF, XGBoost	The accuracy of each algorithm surpassed 90%, while the fusion model achieved an accuracy of 99%	2024	Feng et al. [47]
Milk	Adulteration detection	Hyperspectral imaging	LR ^31^, DT, SVM, LDA ^32^	LDA obtained 100% validation accuracy	2025	Aqeel et al. [116]
Quality assessment						
Milk	Prediction of subclinical mastitis	Daily milk production, fat, protein, casein, lactose, pH, urea, somatic cell count, differential somatic cell count, beta-hydroxybutyrate, electrical conductivity, rennet coagulation time, curd firmness 30 min after rennet addition	Generalized Linear Models, SVM, RF, Neural Network	The neural network achieved the highest accuracy of 0.754	2023	Bobbo et al. [41]
Milk	Prediction of subclinical mastitis	Near-infrared spectra	PLS-DA, RF, SVM	The precision of SVM in detecting non-mastitis milk reached 0.81	2024	da Silva Pereira [65]
Milk	Prediction of subclinical mastitis	Daily milk yield, fat percentage, protein percentage, lactose percentage, milk urea concentration, somatic cell score	Dummy classifier, Logistic Regression, DT, SVM, GNB, KNN		2024	Satoła and Satoła [40]

^1^ NMS: Nonfat Milk Solid; ^2^ AM1: Aflatoxin M1; ^3^ AE: Auto-encoder; ^4^ KNN: K-Nearest Neighbors; ^5^ LOF Local Outlier Factor; ^6^ COF: Connectivity-Based Outlier Factor; ^7^ SO-GAAL: Single-Objective Generative Adversarial Active Learning; ^8^ BN: Bayesian Network; ^9^ MLP: Multilayer Perceptron; ^10^ RASFF: Rapid Alert System for Food and Feed; ^11^ WHO: World Health Organization; ^12^ GEMS: Global Environmental Monitoring System; ^13^ DT: Decision Trees; ^14^ SVM: Support Vector Machines; ^15^ GNB: Gaussian Naive Bayes; ^16^ CNN: Convolutional Neural Networks; ^17^ XGBoost: Extreme Gradient Boosting; ^18^ PPC: Post-Pasteurization Contamination; ^19^ RF: Random Forest; ^20^ MMI: Multimodel Inference; ^21^ DFFNN: Deep Feed-Forward Neural Network; ^22^ GB: Gradient Boosting; ^23^ PLS-DA: Partial Least Square Discriminant Analysis; ^24^ KAN: Kolmogorov-Arnold Networks; ^25^ VCPA-PLS: Variable Combined Cluster Analysis Partial Least-Squares; ^26^ RF-PLS: Randomized Frog Hopping Partial Least-Squares; ^27^ BOSS-PLS: Bootstrap Flexible Shrinkage Variable Selection Partial Least Squares; ^28^ PLSR: Partial Least Square Regression; ^29^ SVR: Support Vector Regression; ^30^ LightGBM: Light Gradient Boosting Machine; ^31^ LR: Logistic Regression; ^32^ LDA: Linear Discriminant Analysis.

### 4.2. Identification of Microbiological Hazards

Bacterial growth in food products may result in contamination and has the potential to instigate foodborne infection when pathogen-contaminated foods are consumed [117]. Globally, contamination presents a substantial risk to public health and well-being: the Center for Disease Control and Prevention (CDC) reports that, annually, approximately forty-eight million people in the United States experience foodborne illnesses caused by inadequate food handling and preparation procedures [118]. Therefore, guaranteeing the safety of food via the timely and precise identification of pathogenic bacteria presents a fundamental challenge for the food supply chain [119]. Al et al. [75] employed ML models (such as ANN, RF, Support Vector Regression (SVR), and Multiple Linear Regression) to analyze the growth of *E. coli* O157:H7 at different temperatures in uncooked ground beef treated with cocktail inoculum. Detection of *E. coli* was based on ISO 16654:2001. The RF model exhibited the highest levels of performance in predicting microbial growth with a coefficient of determination (R^2^) of 0.98, followed by ANN (0.96), SVR (0.85), and Multiple Linear Regression (0.66). An existing dataset of bacterial spoilage indicators (*Pseudomonas* spp.) was used by Murphy et al. [107], acquired from samples of pasteurized milk taken from 23 processing sites. Multimodel Inference (MMI) and RF algorithms identified several factors which correlate with Post-Pasteurization Contamination (PPC). These factors (such as cleaning and sanitation procedures, manufacturing practices, type of container, in-house product assessment, and the presence of a quality control department) can be used to identify potential areas of action for minimizing cases of PPC.

Alshejari et al. [42] created a stacking-based ensemble prediction approach to determine the total viable number of microorganisms in samples of beef filet via multispectral imaging (Table 2). Two advanced clustering-based neuro-fuzzy network prediction models were developed: the first used information concerning average reflectance values, and the second utilized the standard deviation of pixel intensity per wavelength. The performance of each neuro-fuzzy model was evaluated against conventional algorithms (such as MLP, SVM, PLS) and revealed that the neuro-fuzzy models (R^2^ = 0.974 and 0.982) produced the highest levels of performance when compared with a wavelet neural network (R^2^ = 0.974), MLP (R^2^ = 0.967), SVM (R^2^ = 0.965), and PLS (R^2^ = 0.965).

Bacterial species detection in food matrices is a demanding task. The plate counting method of bacterial detection is reliable but has a tedious workflow, while the detection by Enzyme-Linked Immunosorbent Assay (ELISA) requires trained personnel and antibodies of high cost [120,121]. An alternative detection method for overcoming these shortcomings are the sensor arrays which analyze the structure and composition of bacteria, obtaining multiplex responses with high sensitivity [122]. Sensor arrays are based on nanomaterials, such as conjugated polymers, quantum dots, gold nanoparticles, carbon nanomaterials, etc. [123,124,125,126]. In response to the traditional approaches for identifying multiple pathogenic bacteria in contaminated milk (plate counting and ELISA), Wang et al. [60] developed a non-specific optical sensor array using two-dimensional nanoparticles and fluorescence-labeled single-stranded DNA. This study analyzed several bacteria cultures (*E. coli*, *Listeria innocua*, *Salmonella enterica*, *Staphylococcus aureus*, *Shigella sonnei*, *Bacillus cereus*, *Lactococcus lactis*, and *Pseudomonas fluorescens*) and employed the classification algorithms PLS-DA, KNN, RF classifier, Support Vector Classifier (SVC), and two Artificial Neural Networks: MLP, and Kolmogorov–Arnold Networks (KAN). In the testing sets, SVC, KNN, and the RF classifier exhibited elevated levels of accuracy (between 80% and 90%), while the MLP and KAN neural networks demonstrated 93.8% accuracy (following a 30 min incubation period). It should be noted that a 120 min incubation period increased the accuracy of the MLP neural networks to 98.4%.

A study conducted by Jia et al. 2024 [91] utilized paper chromogenic array sensors with a deep feed-forward neural network (DFFNN) to detect *Salmonella Enteritidis* and *E. coli* O157:H7 within a high level of background microflora in shredded cheddar cheese. This approach facilitated the accurate detection of *S. Enteritidis* and *E. coli* O157:H7 in monocultures and cocktail cultures and was able to identify them among a significant level of background microflora (~7.5 log CFU/g) at accuracies ranging between 72 ± 11% and 92 ± 3%. Additionally, this method effectively identified *S. Enteritidis* and *E. coli* O157:H7 at concentrations as low as 1 log CFU/g (within one day) and with an accuracy of 72 ± 11%. Jia et al. [92] employed the same approach (paper chromogenic array sensors and a DFFNN) to concurrently detect *Listeria monocytogenes*, *Salmonella* spp., and *E. coli* O157:H7 in chicken at levels as low as 1 log CFU/g and with an accuracy of more than 90%. Yang et al. 2022 [127] combined a paper chromogenic array with a neural network to identify viable pathogens in the presence of background microflora and microbial spoilage in seafood (via volatile organic compound sensors). The model pathogen and spoilage bacteria employed in the same study were *Morganella morganii* and *Shewanella putrefaciens*. Additionally, it evaluated microbial detection via monoculture and cocktail multiplex detection. The initial accuracy assessments were conducted on standard media and validated on seafood samples (such as cod and salmon) containing pathogenic and spoilage bacteria and background microflora. The neural network was trained on a digitized Red, Green, Blue (RGB) image dataset from the paper chromogenic array images. This approach effectively identified microorganisms from microflora (with and without the prevalent spoilage microbe) and *S. putrefaciens* in seafood with an accuracy level of between 90% and 99%. Potentially, this technique can enhance smart packaging by accomplishing nondestructive pathogen food monitoring without additional procedures such as enrichment, incubation, or sample preparation.

Xu et al. [110] created an integrated ML-based nanogap-assisted Surface-Enhanced Raman Scattering (SERS)/Polymerase Chain Reaction (PCR) biosensor that enables the identification of *Staphylococcus aureus* in milk samples. The bimetallic Au/Ag FL nanoflowers, which have been modified with iodide ions and magnesium sulfate, generate “hot spots” that amplify the Raman signals of *S. aureus’* main gene target. The analysis used three univariate screening algorithms for spectral prediction modeling: The study compared Variable Combined Cluster Analysis Partial Least-Squares (VCPA-PLS), Randomized Frog Hopping Partial Least-Squares (RF-PLS), and Bootstrap Flexible Shrinkage Variable Selection Partial Least Squares (BOSS-PLS) and found BOSS-PLS to be superior with an Rp value of 0.967. The proposed SERS/PCR biosensor proved an efficient and cost-effective alternative to traditional bacterial detection methods.

Electronic nose (e-nose) is a non-destructive and cost-effective technique with increased sensitivity used in food quality and safety monitoring. E-nose uses sensor arrays to detect the effects of odorants in the headspace of samples, simulating the human olfactory system. By applying e-nose, a “fingerprint” of each component in the sample is obtained, which, combined with ML algorithms, provides important information about the sample [128]. One study developed an innovative hybrid model consisting of RF, CNN for local feature extraction, and Gated Recurrent Unit (GRU) for global feature extraction [73]. By employing data from eleven e-nose sensors (including ammonia, hydrogen, and hydrogen sulfide sensors), this hybrid model was able to differentiate between fresh and decayed beef (from twelve different cuts) with higher precision (up to 0.9977) against other ML models such as SVM (0.9864), KNN (0.9887), and CNN (0.9932).

**Table 2:** Applications of machine learning algorithms in meat, meat products, and eggs.

Product	Purpose of Study	Data	Machine Learning Algorithm	Output	Year	Reference
Microbiological hazards						
Milk and beef	Detection of *Escherichia coli* O157:H7	Surface-enhanced Raman scattering–based lateral flow assay	XGBoost ^1^	Successfully forecast *E. coli* in samples spiked with 10 CFU/mL	2020	Yan et al. [85]
Beef	Prediction of total viable counts of microorganisms	Multispectral imaging (wavelength attributes)	Neuro-fuzzy model MLP ^2^, SVM ^3^, PLS ^4^	The neuro-fuzzy model achieved the highest accuracy of 0.982	2023	Alshejari et al. [42]
Meat, eggs, shellfish, dairy products, infant formula, etc.	Identification of parameters associated with the occurrence of *Salmonella* spp.	*Salmonella* spp. occurrence based on: product, region, and stage	RF ^5^	Accuracy achieved 77.2%	2023	Rodríguez et al. [108]
Meat, eggs, dairy products, processed food salad etc.	Identification of potential sources of *Campylobacter* spp.	*Campylobacter* spp. occurrence based on product, region, and stage data	RF	Accuracy achieved 83.1%	2024	Sacristán et al. [109]
Eggs, milk, meats, bakery products etc.	Prevention of foodborne *Salmonella* outbreaks	Food surveillance data (month, longitude, latitude, area, food prevalence, food categories)	Tree regression, RF, GB ^6^	RF and GB (R^2^ = 0.55) outperformed the tree regression algorithm (R^2^ = 0.42)	2024	Garcia-Vozmediano et al. [76]
Chicken	Simultaneous monitoring of multiple pathogens	*Listeria monocytogenes*, *Salmonella*, and *E. coli* O157:H7 detection by paper chromogenic array sensor	DFFNN ^7^	Detection as low as 1 log CFU/g with more than 90% accuracy	2024	Jia et al. [92]
Meat	Spoilage detection	pH sensing patch images dataset	CNN ^8^	Accuracy achieved 0.98	2024	Kadian et al. [129]
Beef	Prediction of *E. coli* O157:H7 growth	Shiga toxin-producing *E. coli* counts	ANN ^9^, RF, SVM, MLR ^10^	RF model exhibited the highest performance (R^2^ = 0.98)	2024	Al et al. [75]
Beef	Spoilage detection	Data from 11 e-nose sensors (including ammonia, hydrogen sulfide, and hydrogen sensors)	SVM, KNN, CNN, hybrid (RF and CNN), hybrid (RF, CNN and GRU ^10^)	The hybrid model of RF, CNN, and GRU achieved 0.9977 accuracy	2024	Surjith et al. [73]
Chemical hazards						
Pork sausages	Monitor residual nitrite concentrations	Hyperspectral imaging (images at the spectral range of 1000–2500 nm)	XGBoost, CATboost ^11^, LightGBM ^12^	XGBoost achieved the highest accuracy (0.999)	2024	Son et al. [86]
Beef	Predict ofloxacin concentration	Thin-layer chromatography-surface-enhanced Raman scattering sensor	BPNN ^13^	A 0.01 ppm sensitivity with an accuracy level of 0.995	2024	Lu et al. [19]
Fraud/adulteration detection						
Beef	Colorant and curing agent adulteration	Diffuse reflectance spectra, color images (RGB components)	AlexNet (with CNN architecture), SVM, Logistic Regression	AlexNet achieved the highest accuracy at 98.84%	2023	Jo et al. [96]
Beef	Detection of adulteration with duck meat	Point discharge microplasma optical emission spectrometer (atomic emission spectra)	LDA ^14^	Accuracy achieved 99.5%	2024	Ren et al. [62]
Quality assessment						
Eggs	Detection of cracked eggs	Images (RGB components)	SVM	Accuracy achieved 98.75%	2020	Haoran et al. [130]
Eggs	Detection of defective eggs	Machine vision system (images dataset)	BiLSTM ^15^	Accuracy achieved 99.17%.	2021	Turkoglu [131]
Eggs	Detection of defective eggs	Machine vision system (images dataset), weight measurements	CNN and RF	Accuracy achieved 94.8%, and R^2^ 96.0%	2023	Yang et al. [72]
Beef	Determine beef quality	RGB images dataset	Deep neural network, LSTM ^16^, GRU ^17^, Bi-GRU ^18^, Bi-LSTM	Bi-LSTM achieved the highest accuracy at 0.989	2024	Büyükarıkan [95]

^1^ XGBoost: Extreme Gradient Boosting; ^2^ MLP: Multilayer Perceptron; ^3^ SVM: Support Vector Machine; ^4^ PLS: Partial Least Squares; ^5^ RF: Random Forest; ^6^ GB: Gradient Boosting; ^7^ DFFNN: Deep Feed-Forward Neural Network; ^8^ CNN: Convolutional Neural Networks; ^9^ ANN: Artificial Neural Networks; ^10^ MLR: Multiple Linear Regression; ^11^ CATboost: Category Boosting; ^12^ LightGBM: Light Gradient Boosting Machine; ^13^ BPNN: Back Propagation Neural Network; ^14^ LDA: Linear Discriminant Analysis; ^15^ BiLSTM: Bi-Directional Long-Short-Term Memory; ^16^ LSTM: Long Short-Term Memory; ^17^ GRU: Gated Recurrent Unit; ^18^ Bi-GRU: Bi-Directional Gated Recurrent Unit.

Meat spoilage can be easily and accurately detected by measuring the pH value due to the methylamines released during bacterial decomposition. According to reports, fresh meat should have a pH between 5.5 and 6.2, while a pH higher than 6.7 indicates unsafe meat. To detect meat spoilage, Kadian et al. [129] developed a microneedle-based colorimetric pH sensing patch. The sensing patch proof-of-concept study utilized a smartphone to determine the pH levels of spoiled and unspoiled meat samples. A CNN classified the images according to their pH values with an accuracy of 0.98.

Evaluation of the various food chain stages (preharvest, processing, and post-harvest) is necessary to control microbiological hazards [132]. Two studies from Sacristán et al. [109] and Rodríguez et al. [108] used data from the Spanish Agency for Food Safety and Nutrition to identify and assess the occurrence of *Campylobacter* spp. and *Salmonella* spp., respectively, in the Spanish food chain employing the RF algorithm.

In the study by Sacristán et al. [109], the RF algorithm showed an accuracy of 83.1%. The results indicated that the presence of Campylobacter spp. was influenced by three variables: primarily product, followed by region and stage of production. Meat products (particularly poultry and sheep) displayed the highest probability of *Campylobacter* spp. occurrence during the initial, intermediate, and final stages of the food chain, and its presence in the final stage (wholesale and retail) suggests potential consumer exposure to bacteria. Rodríguez et al. [108] employed the RF algorithm, achieving an accuracy of 77.2% (for the model with resampling). The study analyzed how product, region, and stage impacted the presence of *Salmonella* spp. and found that the primary determining product was meat while the most crucial stage was identified as the slaughterhouse. Pig and poultry meats displayed the highest values and *Salmonella* spp. probability was defined as high at the initial and final stages (but not in the intermediate stage). The presence of *Salmonella* spp. in the final stage of the food chain (retail) is particularly concerning as it can cause outbreaks of salmonellosis among the exposed population. Garcia-Vozmediano et al. [76] used food safety audit data from different databases combined with ML algorithms (tree regression, RF, and GB) to predict spatiotemporal patterns of salmonellosis in northwestern Italy. Several factors (including the occurrence of *Salmonella* spp. in food, spatial characteristics, and efficient monitoring of milk, fruits, vegetables, and meat from pigs) contributed to the predictive power of the models. RF and GB (R^2^ = 0.55) outperformed the tree regression algorithm (R^2^ = 0.42). This study demonstrated the effectiveness of combining data from human and veterinary health services in developing predictive models of salmonellosis incidence in humans. In addition, it has been confirmed that such an approach can provide early warning to consumers and mitigate the public health impact of foodborne illnesses.

### 4.3. Identification of Chemical Hazards

One of the significant chemical hazards posed by consuming ASFs is the presence of antibiotic residues. In livestock and poultry, antibiotics are frequently used to prevent and control infectious diseases. Nevertheless, overuse of antibiotics results in their residues in food, which enter the body through food consumption [133] and can lead to chronic toxicity and antibiotic resistance [134]. The detection of antibiotics in food can be performed using microbiological methods, High-Performance Liquid Chromatography (HPLC), ELISA, and flow cytometry immunochromatography, although these processes have several limitations such as low sensitivity (microbiological methods, ELISA), requirement of trained operators, and tedious sample pre-treatment (HPLC) [135,136,137,138,139]. Using optical biosensors combined with ML algorithms is a promising alternative with several advantages, such as strong anti-interference ability, fast and cost-effective detection, and on-site application [140]. Zhou et al. [112] combined an optical immunosensor with polystyrene nanoparticles with PLS regression to simultaneously detect the presence of chloramphenicol, kanamycin, and neomycin in milk. The optical immunosensor detected antibiotics in a broad linear range from pg/mL to ng/mL within 30 min and exhibited overall accuracy levels above 99%. Another method developed for the detection of antibiotics is the combination of Thin-Layer Chromatography (TLC), SERS spectroscopy, and the PCA-Back Propagation Neural Network by Lu et al. [19]. The method was applied to detect the quinolone antibiotic ofloxacin in beef. The beef juice was cast directly onto the diatomite TLC plate for separation and detection. The sensitivity was recorded at 0.01 ppm, and the process attained an accuracy level 0.995. Liu et al. [113] created a quasi-ratiometric fluorescent probe for detecting the fluoroquinolone antibiotic levofloxacin in milk. A Europium-based metal–organic framework was designed and paired with levofloxacin’s blue autofluorescence to build a detection system with a large linear detection range (0.01–175 μM) and a low detection limit (4.53 nM). A hierarchical clustering algorithm was developed in combination with a smartphone test strip platform to speed up the detection process. The system produced excellent recovery rates (101.7–103.4%) while minimizing deviations. Liu et al. [114] employed SERS and ML to identify the quinolone drugs norfloxacin and ciprofloxacin in milk. A prediction model that utilized various ML methods, such as SVR, RF, and XGBoost, was used to achieve an R^2^ value of 0.996 with a sensitivity threshold of 10 ppb.

Another important chemical hazard is the presence of mycotoxins, which are secondary metabolites of phytopathogenic fungi that have detrimental effects on both human and animal health. They frequently appear as foodborne contaminants that are introduced during the manufacturing or storage of food [141]. A study performed by Aggarwal et al. [111] applied array sensing with Alizarin Red S and cystamine-derived carbon dot in combination with an XGBoost model for the detection of mycotoxins (citrinin, aflatoxin B1, and ochratoxin A). The method was applied in several foods including milk, obtaining 100% accuracy and detection at low concentrations (10 pmol of mycotoxin).

**Figure 9:**
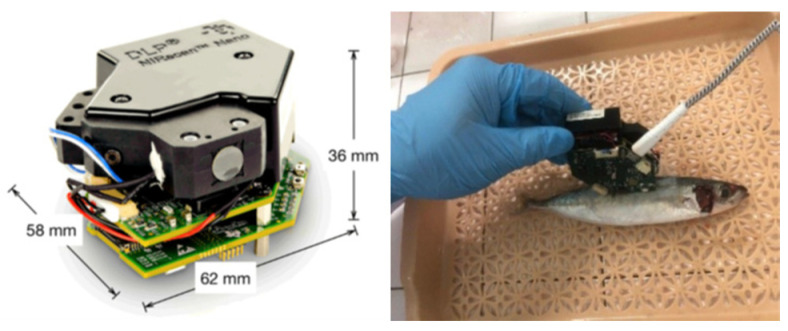
The NIR device (**left**). An example of spectrum measurement on fish (**right**). From Ninh et al. [58].

Residual nitrites present serious health risks in cured meat products. The use of nitrites is vital for creating desirable quality attributes, including flavor enhancement, color development, oxidative stability, and microbiological safety. Although 30% to 60% of nitrites react with the lipids and proteins in the muscles, 5% to 20% of the nitrites remain residual nitrites in the final product. These residual nitrites may react with secondary amines to produce carcinogenic nitrosamines [142]. Son et al. [86] evaluated the combination of hyperspectral imaging and ML algorithms (XGBoost, CATboost, and LightGBM) to predict and monitor the concentrations of residual nitrites in emulsified pork sausages. The hyperspectral imaging measurements were captured through images of the cross-section and lateral sides of sausage samples in line scan mode (with a spectral range of 1000–2500 nm). The resulting analysis indicated that increased nitrite concentrations could affect the protein matrix and hydrogen-bonding capacities, resulting in increased reflectance at approximately 1080 nm and 1280 nm. The accuracy levels of the ML models were XGBoost (0.999), CATboost (0.998), and LightGBM (0.990). Using urea and ice to preserve seafood causes an endothermic reaction that decreases the water temperature as the urea dissolves, extending the fish’s freshness. As a result, seafood is vulnerable to urea contamination. One study [58] investigated the classification of fish samples (mackerel, tuna, and pompano) as safe or unsafe (based on their urea content) through a combination of near-infrared (NIR) spectroscopy (Figure 9) and ML algorithms (DT, KNN, SVM, CNN, and XGBoost) (Table 3). CNN showed the highest accuracy at 83.9%, followed by XGBoost at 81.6%, DT at 79.6%, KNN at 78.9%, and SVM at 78.1%.

**Table 3:** Applications of machine learning algorithms in fish.

Product	Purpose of Study	Data	Machine Learning Algorithm	Output	Year	Reference
Microbiological hazards						
Cod, salmon	Detection of viable pathogens	Paper chromogenic array images dataset for *Morganella morganii*, *Shewanella putrefaciens* detection	Neural network	Accuracy reached 90% to 99%	2022	Yang et al. [127]
Chemical hazards						
Fish	IoT ^1^ sensors for formaldehyde detection, fish freshness detection	Formaldehyde sensor ppm level concentration data, images dataset	CNN ^2^, DNN ^3^	Accuracy reached 99.02%	2024	Harish et al. [143]
Mackerel, tuna, and pompano species	Classification of fish into safe and unsafe based on urea content	Near-infrared spectroscopy data	DT ^4^, KNN ^5^, SVM ^6^, XGBoost ^7^, CNN	CNN achieved the highest accuracy at 83.9%	2024	Ninh et al. [58]
Fish	Determine the freshness and formaldehyde	Formaldehyde sensor data, Images dataset	CNN	Accuracy reached 98.2%	2024	Joy et al. [144]
Tuna	Assessment of histamine levels	Near-infrared spectroscopy data	PLSR ^8^, RF ^9^, SVM	SVM binary and multiclass models achieved the highest accuracy at 100% and 93% respectively	2025	Currò et al. [43]
Fishery products	Detection of biogenic amines	LA-DBD-TLC-MS ^10^ data	PCA ^11^, RF, SVM, MLP ^12^	MLP achieved 100% accuracy and detection limit of 0.230 pg/mm^2^	2025	Zhang et al. [145]
Quality assessment						
Fish	Evaluation of fish freshness	Images dataset	KNN, SVM, LR ^13^, RF, ANN ^14^	Accuracy ranged from 99.6 to 100%	2023	Yasin et al. [48]
Salmon and sablefish filets	Quality assessment	Visible near-infrared, short-wave infrared reflectance, and fluorescence spectroscopy data	SOM ^15^, LDA ^16^, QDA ^17^, KNN, RF, SVM, linear regression	The highest accuracy at 95% was obtained from the combination of three spectroscopy modes with LDA	2023	Kashani Zadeh et al. [59]
Indian sardinella, yellowfin tuna	Quality evaluation	Images dataset	Neural Network architectures FishNET-S and FishNET-T	FishNET-S achieved an accuracy of 84.1% and FishNET-T 68.3%	2023	Jayasundara et al. [146]
Sea bass	Freshness detection	Raman spectra data	PLS-DA^18^, SVM, CNN	CNN achieved the highest accuracy at 90.6%	2023	Wang et al. [39]
Fish	Real-time freshness detection	Temperature, total viable count, total volatile basic nitrogen, K-value, electronic nose, gas chromatography-mass spectrometry, sensory analysis data	BP ^19^, GA-BP ^20^, RBF ^21^, ELM ^22^	RBF neural network achieved the highest R^2^ value at 0.9994	2024	Cui et al. [94]
Mackerel, tuna, and pompano Species	Evaluating fish quality based on histamine content	Near-infrared spectroscopy data	DT, KNN, SVM, XGBoost, CNN	CNN achieved the highest accuracy at 93%	2024	Ninh et al. [57]
Fish	Real-time evaluation of balsa fish freshness	Colorimetric sensor array data	PLSR, RF	RF achieved a higher correlation coefficient of prediction value (0.981) than PLS (0.877)	2025	Cao et al. [74]

^1^ IoT: Internet of Things; ^2^ CNN: Convolutional Neural Networks; ^3^ DNN: Dense Neural Networks; ^4^ DT Decision Trees; ^5^ KNN: K-Nearest Neighbors; ^6^ SVM: Support Vector Machines; ^7^ XGBoost: Extreme Gradient Boosting; ^8^ PLSR: Partial Least Square Regression; ^9^ RF: Random Forest; ^10^ LA-DBD-TLC-MS: Laser Ablation Dielectric Barrier Discharge Thin-Layer Chromatography-Mass Spectrometry; ^11^ PCA: Principal Component Analysis; ^12^ MLP: Multilayer Perceptron; ^13^ LR: Logistic Regression; ^14^ ANN: Artificial Neural Networks; ^15^ SOM: Self-Organized Maps; ^16^ LDA: Linear Discriminant Analysis; ^17^ QDA: Quadratic Discriminant Analysis; ^18^ PLS-DA: Partial Least Squares-Discriminant Analysis; ^19^ BP: Back Propagation; ^20^ GA-BP: Genetic Algorithm Back Propagation; ^21^ RBF: Radial Basis Function; ^22^ ELM: Extreme Learning Machine.

Histamine is a toxic metabolite created by histamine-producing bacteria during the spoilage and fermentation of fish (and its associated products). It is heat-stable which means that traditional industrial and domestic processes (such as cooking) do not decrease its presence in foodstuffs. Additionally, it does not produce any observable changes in the product; therefore, increasing consumer awareness of its presence may be ineffective in reducing the consumption of histamine-contaminated fish. Currò et al. [43] conducted research concerning histamine contamination in samples of tuna by adopting an integrated approach which combined NIR spectroscopy with innovative ML (Modified Partial Least Squares Regression (MPLS) and SVM).

In this study, samples of tuna were treated with four histamine concentrations (0, 50, 150, and 200 mg/kg). For the quantification prediction, the MPLS (which used the full spectrum) exhibited good (R^2^CV = 0.88) and approximate (R^2^P = 0.74) predictive performance when estimating the amount of histamine present in the samples. The SVM classification models—both binary (present/absent) and multiclass (four levels)—exhibited prominent levels of accuracy (100% and 93%, respectively) and effectively identified classes with concentrations above 100 mg/kg. Zhang et al. [145] developed a new LA-DBD-TLC-MS device that automates the detection of histamine, tyramine, putrescine, cadaverine, spermine, and phenylethylamine in fishery products. The system merges a diode laser with DBD plasma ionization and mass spectrometry. The detection limit of the LA-DBD-TLC-MS device reached 0.230 pg/mm^2^, which matches the detection capabilities of high-performance liquid chromatography-mass spectrometry. ML techniques including PCA, RF, SVM, and MLP proved effective for species categorization and spoilage evaluation while MLP achieved perfect accuracy at 100%.

In fish and seafood, formaldehyde has a natural formation pathway; however, it can be added illegally to extend the shelf life. Harish et al. [143] and Joy et al. [144] conducted research regarding the presence of formaldehyde in fish and the detection of fish freshness. Each study combined IoT sensor technology with neural networks and observed accuracy levels of 99.02% and 98.2%, respectively (Table 3).

### 4.4. Fraud/Adulteration Detection

Misleading food consumers for financial reasons is known as economically motivated adulteration. Compared to other conventional threats, this practice significantly exacerbates food safety problems because the contaminants are often unconventional and have unknown health effects. Milk is one of the most frequently targeted food commodities in food fraud. Fraudsters used nitrogen-rich substances to tamper with milk protein, based on protein specifications, in order to make the protein values seem genuine [147]. The 2008 melamine-tainted infant formulae outbreak caused acute kidney failure, nephrolithiasis, and other abnormalities of the urinary system in infants and young children, illustrating the seriousness of the human cost of food adulteration [148,149,150]. During the 2013 horsemeat scandal in Europe, it was discovered that food labeled as beef actually contained unreported horse meat [151]. Therefore, an efficient protocol is required to identify adulterants that have not yet been discovered. Chung et al. [115] employed an ensemble ML model consisting of ExtraTrees and XGBoost to identify unprecedented adulteration without seeking for specific ingredients, that is, in a non-targeted method. The study used varying concentrations of potassium sulfate, potassium dichromate, citric acid, sodium citrate, ammonium sulfate, melamine, urea, lactose, glucose, sucrose, maltodextrin, fructose, water, whole milk powder, skim milk powder, starch, soy milk, and trisodium citrate to create adulterated samples for testing. The dataset utilized for this study consisted of compositional data of raw milk (fat, protein, non-fat solid, total solid, lactose, relative density, freezing point depression, and acidity) and Fourier Transform Infrared (FTIR) spectra. The proposed ensemble M model achieved an accuracy level of 0.9924.

A study conducted by Feng et al. [47] employed ML models which used Raman spectra to identify dairy fraud. The study combined a LightGBM, RF, and XGBoost with the CARS algorithm as a feature extraction method. The accuracy of each of the adopted algorithms exceeded 90% when differentiating between dairy brands. Additionally, following synergistic combination, the fusion model attained an accuracy level of 99%. Aqeel et al. [116] investigated methods to detect milk adulteration, employing both destructive and nondestructive approaches. The destructive method utilized the Lactoscan system to measure milk properties such as solids-not-fat, density, fat, lactose, conductivity, protein, temperature, and pH. Hyperspectral imaging in the 397–1,003 nm range captured spectral signatures for the nondestructive method. The sample set contained 50 pure samples and 400 samples contaminated with salicylic acid, boric acid, glucose and formalin which underwent processing through radiometric correction and picture scaling methods. One-vs-One LDA achieved perfect validation accuracy of 100% which made it stand out as the most effective algorithm among Logistic Regression, DT, SVM, and LDA. Hyperspectral imaging and ML algorithms performed better than conventional methods in detecting milk adulteration.

Jo et al. [96] developed a deep-learning model based on diffuse reflectance spectroscopy and color images for the identification of counterfeit beef which had been modified to resemble fresh product. The AlexNet model (which utilizes CNN) was applied to the samples using a spectral range of 344–1040 nm and achieved a classification accuracy of 98.84%. In order to detect beef adulteration and enable on-site food freshness assessments, Ren et al. [62] integrated a portable device based on a point discharge microplasma optical emission spectrometer with ML. This device was incorporated into two modular injection units (headspace solid-phase microextraction and headspace purge) to facilitate sample evaluation. This approach was effective at detecting the adulteration of beef samples (containing variable amounts of duck meat) with an accuracy level of 99.5% using LDA.

### 4.5. Food Quality Assessment

#### 4.5.1. Milk Quality

Subclinical mastitis in cows may impact the nutritional composition of milk and compromise consumer safety. It does not instigate any observable changes in the udder or the milk; therefore, it is more challenging to identify than clinical mastitis. A study conducted by da Silva Pereira et al. [65] examined the use of NIR spectroscopy by utilizing a portable spectrometer to identify milk which had been contaminated with subclinical mastitis. PCA, PLS-DA, RF, and SVM were employed to analyze the NIR spectra. The PCA disclosed that the clustering of mastitis and non-mastitis milk samples correlated with lactose content fluctuations, which aligns with the PLS-DA (which achieved an accuracy of 78%). RF and SVM both attained accuracy levels of 62% in detecting mastitis milk. However, it should be noted that RF displayed higher levels of sensitivity (recall) of 78% for detecting mastitis, while SVM achieved elevated levels of accuracy for detecting non-mastitis milk (81%). Additionally, the use of an Isolation Forest to eradicate outliers enhanced the performance of RF and SVM models based on NIR spectra and improved precision by up to 25%. The results of this research indicate that the portable spectrometer shows potential as a screening method for the detection of mastitis milk samples in the dairy industry. Satoła and Satoła [40] employed ML to classify Polish Holstein–Friesian cows as healthy or at risk of subclinical mastitis. Their research utilized a dataset containing information gathered during routine milk procedures such as daily milk yield; fat, protein, and lactose percentage; milk urea concentration; and somatic cell count (SCC). They created ensemble ML models (specifically bagging, boosting, stacking, and super-learner) and single ML models (such as SVM, LR, GNB, KNN, and DT). GB (0.767) and SVM (0.767) exhibited the highest levels of accuracy for the training datasets, followed by Super-Learner LR (0.765), LR (0.765), RF (0.763), Voting Ensemble RFC (0.754), GNB (0.753), KNN (0.736), and DT (0.685). Bobbo et al. [41] combined a dataset containing routine milk recording procedures and climatic data with ML algorithms (Generalized Linear Model (GLM), SVM, RF, and neural networks) to predict the existence or nonexistence of subclinical mastitis among Italian Mediterranean buffalo. When examining the data, the most relevant animal-based information concerned Somatic Cell Score, differential SCC, electrical conductivity, and milk production. Among the climate data, the most useful features concerned temperature and relative humidity. The SVM was the most effective method for predicting an elevated or reduced somatic cell count at the subsequent test-day record in the validation set; therefore, this approach was employed to appraise the contribution of each characteristic to the most appropriate model. The neural network was the most effective at making predictions on the test set. Splitting the original dataset by record obtained the following levels of accuracy: GLM (0.752), SVM (0.739), RF (0.740), and the neural network (0.754).

#### 4.5.2. Meat Quality

The quality and color of meat are influenced by various factors, including temperature, microbial activity, humidity, and freezing and thawing. To assess meat quality and safety, both subjective and objective evaluation methods are employed [152]. Since subjective evaluation relies on appearance to gauge quality, replicating results can be challenging [153]. Laboratory tests are utilized to determine several meat characteristics in objective evaluation, including pH, color, temperature, and microbial testing [154]. These evaluation methods can result in significant food waste and are time-consuming. Alternatively, color analysis can be conducted using computer vision. The researchers of [95] carried out a non-destructive assessment of beef quality (fresh, half-fresh, and spoiled) based on RGB images obtained for color analysis. This was accomplished by combining learning-based global and handcrafted color features through the LSTM, bi-directional long short-term memory (Bi-LSTM), GRU, and bi-directional Gated Recurrent Unit (Bi-GRU) of the neural networks. The highest levels of accuracy (0.989) were achieved via an amalgamation of the following features: Visual Geometry Group 16 (VGG16); hue, lightness, and saturation (HLS); hue, saturation, and value (HSV); RGB; and Bi-LSTM neural network.

#### 4.5.3. Fish Quality

Ninh et al. [57] combined NIR spectroscopy with ML techniques to classify fish samples (mackerel, tuna, and pompano) as either safe or unsafe according to whether their histamine content exceeded the permissible limit of 100 ppm. When combined with an optimized CNN, a feature extraction technique (using pre-processed NIR spectra and their second derivatives) surpassed the accuracy of traditional ML classifiers (DT—87.2%, KNN—83.2%, SVM—86.3%, and XGBoost—90.3%) by exhibiting an accuracy of 93.1%. To facilitate the efficient classification of the quality of sea bass filets, Wang et al. [39] employed a CNN to model Raman spectra data. This study concluded that ANOVA was a suitable method for extracting the Raman spectral features and that the most effective model for assessing the freshness of sea bass fillets was the feature-selected CNN model, which produced a classification accuracy of 90.6%. Kashani Zadeh et al. [59] combined data fusion of visible near-infrared (VIS-NIR) with Short Wave Infrared (SWIR) and fluorescence spectroscopy data to classify the condition of salmon and sablefish as either fresh or spoiled. This research utilized several ML algorithms (including PCA, SOM, LDA, QDA, KNN, RF, SVM, and LR). The results demonstrated that multi-mode spectroscopy achieved 95% accuracy and enhanced the precision of single-mode spectroscopies as follows: FL by 26%, VIS-NIR by 10%, and SWIR by 9%.

Yasin et al. [48] used KNN, SVM, ANN, RF, and LR ML algorithms to classify the freshness of fish by employing an image dataset of deceased fish which had been categorized as either fresh or stale. Following this, SqueezeNet and InceptionV3 algorithms were utilized for the feature extraction process. The results of the study revealed that the SVM, ANN, and LR models result in an accuracy rate of 100% for each ML algorithm while accuracy for RF was 99.7% and KNN was 99.9%. Jayasundara et al. [146] employed two Convolutional Neural Network architectures (FishNET-S and FishNET-t) to evaluate the quality of Indian Sardinella and Yellowfin Tuna via RGB images taken by smartphone cameras. The selected CNN architectures employ two different methodologies: FishNET-S is founded on the VGG-16 with the addition of a Block Attention Module (BAM) which directs the model to learn the physical features associated with evaluating the quality of a fish (specifically via the area around the eye region); contrastingly, FishNET-T conducts a color decomposition analysis (based on saturation, hue, and transformations in intensity) and forwards the saturation and hue elements to the CNN to determine the quality grade of each fish via its flesh. This research reveals that FishNET-S has attained an accuracy level of 84.1% and FishNET-T has an accuracy level of 68.3%. A comparison analysis of these two architectures (conducted via standard ML and advanced deep learning models) concludes that the performance of FishNET-S and FishNET-T is superior to that of alternative models.

A study conducted by Cao et al. [74] created a colorimetric sensor array to facilitate the real-time monitoring of the freshness of balsa fish. This was achieved by loading various acid-base indicators onto Attapulgite/Polyimide Nanofiber Composite Aerogels (ATP/PI NFAs). PCA was utilized for feature extraction and, subsequently, PLS and RF regression models were established via the preferred color characteristic values. The correlation coefficient of prediction (Rp) values are as follows: PLS (0.877) and RF (0.981).

Cui et al. [94] applied Back Propagation (BP), Radial Basis Function (RBF), Genetic Algorithm-BP (GA-BP), and ELM to predict the shelf life of five varieties of marine fish (*Sciaenops ocellatus*, *Epinephelus akaara*, *Trachinotus ovatus*, *Larimichthys crocea*, and *Rainbow trout*). The dataset contained several parameters including temperature, total viable count, total volatile basic nitrogen, K-value, electronic nose-Gas Chromatography-Tandem Mass Spectrometry (GC-Ms/Ms), and sensory evaluation. The R^2^ values were reported as follows: RBF neural network (0.9994), GA-BP (0.9989), ELM (0.9986), and BP (0.9981).

#### 4.5.4. Egg Quality

For both financial and safety reasons, separating defective eggs (i.e., those that are cracked, dirty, or externally contaminated) from quality eggs is a critical issue. One study developed a real-time machine vision system based on deep learning to facilitate the identification of defective eggs [131]. In this procedure, a pre-trained residual network model extracted deep features, which were then supplied to the Bi-LSTM neural network. The effectiveness of this approach was evaluated by presenting a selection of defective and quality eggs to the machine vision system, resulting in an impressive accuracy level of 99.17%. Another study by Haoran et al. [130] utilized SVM to create an egg crack image recognition method. The image of the egg crack was pre-processed to eliminate environmental noise and enhance the efficient extraction of the egg crack area. This process enabled the model to achieve accuracy levels of 98.75%, confirming its recognition and classification capabilities.

## 5. Summary of Findings

The implementation of ML algorithms in ASF food safety procedures has led to substantial improvements in risk assessment while enabling better hazard identification, fraud detection, and quality evaluation. ML models such as neural networks (MLP, LSTM, CNN), SVM, and RF algorithms are frequently applied to large datasets from spectroscopic techniques, imaging methods, and sensor-based monitoring. Neural networks demonstrate superior performance when processing complex imaging and sensor data but present challenges in interpretability, while SVM and RF models provide better interpretability and stronger protection against overfitting. Implementing WHO’s GEMS and the EU’s RASFF databases improved predictive food safety risk monitoring models by enhancing early hazard detection abilities. ML models combined with new technologies like electronic noses and hyperspectral imaging enable quick and precise non-invasive tests of microbiological and chemical hazards, significantly impacting food safety.

The deployment of unsupervised AE networks and Bayesian models for anomaly detection in dairy supply chains represents a significant innovation in early warning systems. ML techniques applied to HACCP monitoring through remote sensor diagnostics like the BEST system demonstrate a transition to automated real-time food safety solutions. New sensor-based techniques such as fluorescence-labeled DNA probes and paper chromogenic arrays have made fast pathogen detection possible for milk, meat, and seafood products. The new detection methods outperform traditional culture-based techniques in terms of speed and sensitivity since neural network models produce pathogen identification classification accuracies above 90%. IoT-connected models show exceptional accuracy when measuring spoilage signals such as volatile organic compounds and formaldehyde in seafood, which marks a shift towards advanced food monitoring systems.

ML applications have a significant impact on food in the detection of chemical hazards and represent a novel path for innovative advancements. ML models, including XGBoost, LightGBM, and CNN, have been integrated with optical biosensors and Raman spectroscopy alongside hyperspectral imaging to achieve near-perfect detection rates for antibiotic residues as well as mycotoxins and nitrite levels. ML-enhanced sensors enable quick multi-contaminant detection which revolutionizes traditional labor-heavy laboratory approaches. Non-targeted ML techniques like ensemble learning and deep-learning spectral analysis now enable fraud detection in dairy and meat products to achieve unprecedented accuracy levels. Portable spectrometers combined with smartphone classifiers make food safety diagnostics more accessible and provide affordable on-site detection options.

Another impactful application of ML is the detection of food adulteration. The melamine contamination of infant formula underscored the necessity for enhanced ML techniques. The ExtraTrees and XGBoost ensemble learning models successfully identified milk adulteration by utilizing compositional data in conjunction with FTIR spectral analysis. Deep learning-based spectral analysis tools have served to detect fraudulent changes in beef products while promoting transparent labeling. ML applications enhance food safety and protect consumers from fraud and health risks which improves the security and reliability of the global food supply chain.

This study demonstrates the capability of ML to revolutionize food safety methods by replacing traditional reactive measures with proactive real-time monitoring systems. ML combined with new digital and sensor technologies now allows fast and precise evaluations throughout the food supply chain starting from production until retail. This study surpasses former literature reviews [6,16,17,25,28,30,155,156] by merging deep learning and big data analysis with sensor-based food safety monitoring and HACCP systems to enhance food safety risk management in ASF. Reviewed studies enhance food safety monitoring through higher accuracy and scalability which results in better proactive management abilities than those offered by traditional risk assessment techniques.

## 6. Categorization of Machine Learning Applications in Food Safety, Limitations, and Future Directions

The field of food safety has been revolutionized by ML which provides versatile applications in multiple areas. Auto-encoder networks and Bayesian models as ML tools in risk assessment help detect hazards early, which enhances both real-time monitoring and regulatory compliance. These applications encounter difficulties due to demanding computational requirements and challenges in data integration. Fluorescence-labeled DNA probes and chromogenic sensors enable the quick non-invasive detection of pathogens, which helps minimize contamination risks in microbiological hazard detection. Despite technological progress, these methods still face problems with detecting background microflora and environmental fluctuations.

ML applications help chemical hazard identification processes by combining optical biosensors and Raman spectroscopy to achieve a better detection accuracy of harmful chemicals. Although these methods improve food safety, they depend on expensive technological solutions and regulatory permissions which act as barriers to broad implementation. ML serves as an important tool for detecting fraud and adulteration by utilizing ensemble learning models and spectral analysis approaches to authenticate food goods and combat economic fraud. When detecting various forms of adulterants, these models require big datasets to ensure proper generalization.

Neural networks used in food quality assessments predict both freshness and spoilage which helps to reduce food waste and ensure quality control but model precision suffers due to environmental fluctuations. Smart packaging and storage systems with integrated ML technology and IoT-enabled e-nose sensors deliver real-time monitoring of food quality while improving shelf life. These technological advancements encounter adoption challenges because of their expensive infrastructure requirements. ML advancements in food safety remain limited without further research into cost efficiency and standardization to enable regulatory compliance for expanded use.

ML and sensor-based technologies reveal promise in food safety monitoring but encounter major limitations. The training and validation of ML models depend on extensive high-quality datasets which often reveal inconsistencies and gaps when transferred to various food production systems or regional contexts. Different data sources with chemical contaminants and microbiological hazards create biases which reduce model generalizability. Unsupervised models and advanced neural networks perform exceptionally well at detecting foodborne pathogens and contaminants. Food safety professionals cannot comprehend how results are obtained because the decision-making processes of these technologies stay unclear [73]. The deployment of paper chromogenic arrays and e-nose systems results in quick detection capabilities and high sensitivity but faces practical deployment difficulties because of high costs and scalability challenges and environmental interference in real-world applications [92,157].

Future research needs to prioritize developing standardized datasets accessible to the public and cover a range of food matrices and hazards from various regions. This initiative will boost the adaptability of models across different applications and improve the robustness of predictive analytics in diverse settings. As ML models become more intricate, their development must integrate features to support non-expert users in making well-informed decisions. Future studies ought to investigate ML models that combine spectroscopic methods with e-nose sensor arrays and real-time monitoring systems to enhance accuracy in detecting contamination from both chemical and microbiological hazards. Additionally, research should prioritize the integration of ML and IoT technology to create automated systems for monitoring food safety in real-time. The employment of AI on portable spectrometers, along with hyperspectral imaging and biosensors, allows users to make decisions on-site. Self-learning ML models, such as reinforcement learning, hold the potential to reduce labeled data requirements while enhancing adaptability and predictive accuracy, which will facilitate the cost-effective scaling of ML food safety systems.

## 7. Conclusions

As the landscape and scope of food safety evolve, it is evident that ML can have a positive impact on quality assurance protocols throughout the agri-food sector. To our knowledge, studies that explicitly linked ML with HACCP monitoring in ASFs are limited. The findings of this review emphasize the revolutionary potential for ML to reinforce and enhance existing food safety and HACCP monitoring protocols in the domain of ASFs. However, as supervised and unsupervised ML algorithms continue to augment the detection of anomalies and enhance the risk prediction of foodborne pathogens, proactive monitoring and analysis become increasingly necessary. Adopting spectroscopic techniques, such as NIR, FTIR, and Raman spectroscopy, could complement ML algorithms and facilitate the development of rapid and non-destructive techniques for analyzing Animal-Source Foods. Advancements in sensory technology (including smartphone-based sensors and paper chromogenic arrays) permit the implementation of real-time monitoring techniques for assessing food freshness and detecting pathogens to enhance food safety management. Additionally, machine vision and neural networks provide a rapid and non-destructive alternative to the currently established methodologies. To further enhance food safety, future research should explore the use of real-time monitoring paradigms (involving ML algorithms) to assess data along the entire food supply chain continuously.

## Data Availability

No new data were created or analyzed in this study. Data sharing is not applicable to this article.

## Abbreviations

The following abbreviations are used in this manuscript:

ML	Machine Learning
HACCP	Hazard Analysis Critical Control Points
ASF	Animal-Source Foods
ANOVA	Analysis of Variance
RFE	Recursive Feature Elimination
GA	Genetic Algorithms
LASSO	Least Absolute Shrinkage and Selection Operator
CNN	Convolutional Neural Networks
PLS	Partial Least Squares
PCA	Principal Component Analysis
CARS	Competitive Adaptive Reweighted Sampling
NB	Naive Bayes
DT	Decision Trees
KNN	K-Nearest Neighbors
SVM	Support Vector Machine
LR	Logistic Regression
RF	Random Forest
DA	Discriminant Analysis
GB	Gradient Boosting
GNB	Gaussian Naïve Bayes
LDA	Linear Discriminant Analysis
PLS-DA	Partial Least Squares-Discriminant Analysis
PLSR	Partial Least Squares Regression
XGBoost	Extreme Gradient Boosting
LightGBM	Light Gradient Boosting Machine
CatBoost	Category boosting
ANN	Artificial Neural Networks
SLP	Single-Layer Perceptron
MLP	Multilayer Perceptron
RBNN	Radial Basis Neural Networks
ELM	Extreme Learning Machines
SOM	Self-organizing Map
ART	Adaptive Resonance Theory
LSTM	Long Short-Term Memory
AE	Auto-encoder
AM1	Aflatoxin M1
GEMS	Global Environmental Monitoring System
RASFF	Rapid Alert System for Food and Feed
KAP	Quality Program for Agricultural Products
POPAs	Points of Particular Attention
CDC	Centers for Disease Control and Prevention
PPC	Post-Pasteurization Contamination
ELISA	Enzyme-Linked Immunosorbent Assay
SVC	Support Vector Classifier
SVR	Support Vector Regression
KAN	Kolmogorov–Arnold Networks
DFFNN	Deep Feed-Forward Neural Network
GRU	Gated Recurrent Unit
HPLC	High-Performance Liquid Chromatography
TLC	Thin-Layer Chromatography
SERS	Surface-Enhanced Raman Scattering
NIR	Near-Infrared
MPLS	Modified Partial Least Squares Regression
FTIR	Fourier Transform Infrared
SCC	Somatic Cell Count
GLM	Generalized Linear Model
Bi-LSTM	Bi-Directional Long-Short-Term Memory
Bi-GRU	Bi-Directional Gated Recurrent Unit
VGG16	Visual Geometry Group 16
RGB	Red, Green, Blue
HLS	Hue, Lightness, Saturation
HSV	Hue, Saturation, Value
ATP/PI NFAs	Attapulgite/Polyimide Nanofiber Composite Aerogels
BP	Back Propagation
RBF	Radial Basis Function
GA-BP	Genetic Algorithm-Back Propagation
GC-Ms/Ms	Gas Chromatography-Tandem Mass Spectrometry
NMS	Nonfat Milk Solid
LOF	Local Outlier Factor
COF	Connectivity-Based Outlier Factor
SO-GAAL	Single-Objective Generative Adversarial Active Learning
BN	Bayesian Network
WHO	World Health Organization
KAN	Kolmogorov–Arnold Networks
MMI	Multimodel Inference
MLR	Multiple Linear Regression
BPNN	Back Propagation Neural Network
DNN	Dense Neural Networks
QDA	Quadratic Discriminant Analysis
VIS-NIR	Visible Near-Infrared
SWIR	Short Wave Infrared
LA-DBD-TLC-MS	Laser Ablation Dielectric Barrier Discharge Thin-Layer Chromatography-Mass Spectrometry
VCPA-PLS	Variable Combined Cluster Analysis Partial Least-Squares
RF-PLS	Randomized Frog Hopping Partial Least-Squares
BOSS-PLS	Bootstrap Flexible Shrinkage Variable Selection Partial Least Squares
PCR	Polymerase Chain Reaction
